# Current endoscopic diagnosis treatment strategy for superficial nonampullary duodenal tumours

**DOI:** 10.1186/s40001-022-00940-4

**Published:** 2022-12-14

**Authors:** Aichun Li, Jianwei Shen

**Affiliations:** Department of Gastroenterology, Ningbo Medical Center Lihuili Hospital, Ningbo, 315040 China

**Keywords:** Superficial nonampullary duodenal epithelial tumors, Endoscopic diagnosis, Endoscopic mucosal resection, Endoscopic submucosal dissection, Laparoscopic endoscopic cooperative surgery

## Abstract

**Background:**

Preoperative endoscopic diagnosis and timely treatment are important for the clinical management of sporadically superficial nonampullary duodenal epithelial tumours (SNADETs), including adenoma and adenocarcinoma limited to the submucosal layer.

**Methods:**

This review explores current endoscopic diagnosis and endoscopic resection technology for SNADETs. We compare endoscopic diagnosis accuracy using white light imaging, narrow band imaging, and magnification endoscopy alone or in combination. In addition, we review the current endoscopic resection methods for SNADETs and discuss the limitations and applicable future directions of each technology.

**Results:**

A simple scoring system based on the endoscopic findings of white light imaging or magnified endoscopy combined with image-enhanced techniques was applied for the prediction of the histological grade of SNADETs. Benign or low-grade adenoma can be followed up without biopsy, and high-grade adenoma and adenocarcinoma should be resected by endoscopic mucosal resection (EMR), endoscopic submucosal dissection (ESD), or surgery. EMR frequently leads to a piecemeal resection, while ESD ensures a high en bloc resection rate with a high risk of complications. Covering or closing post-ESD ulcers is an effective strategy to reduce the risk of delayed perforation and bleeding. Laparoscopic endoscopic cooperative surgery is a promising treatment for SNADETs with excellent rates of en bloc resection and a low risk of complications, although it is expensive and requires many specialists.

**Conclusions:**

Early endoscopic diagnosis and optimal treatment selection for SNADETs may improve the poor prognosis of duodenal cancer.

## Introduction

Superficial nonampullary duodenal epithelial tumours (SNADETs), including adenoma and adenocarcinoma limited to the submucosal layer, are rare, but the treatment outcomes for advanced cases are not satisfactory [1]. Reportedly, 4.7% of duodenal adenomas progress to adenocarcinoma, and the risk increases with increasing lesion diameter histological grade [2]. Therefore, it is important to diagnose and treat early stage SNADETs.

Recent studies have mainly focused on the association between endoscopic findings and the histology of lesions with advanced endoscopy techniques, because preoperative biopsy is undesirable for duodenal lesions, because it has poor accuracy and can cause unexpected fibrosis [3]. Furthermore, histological grade is important for clinical management decisions. Low-grade duodenal adenoma (LGA) can be followed up, but high-grade adenoma (HGA) or higher among SNADETs without metastasis should be treated by endoscopic resection, including endoscopic mucosal resection (EMR) and endoscopic submucosal dissection (ESD). Submucosal invasive adenocarcinomas with lymph node dissection should be treated by highly invasive surgery, such as pancreaticoduodenectomy. Therefore, it is feasible to resect and cure duodenal tumours at the precancerous stage with less invasive endoscopic treatment under early endoscopic diagnosis. However, the standard of endoscopic diagnosis and treatments for SNADETs has not been established due to their rarity. In this review, we focused on current endoscopic diagnosis and resection methods for SNADETs.

## Endoscopic diagnosis for SNADETs

The gold standard for gastrointestinal tract tumour diagnosis is preoperative endoscopic biopsy. However, preoperative biopsy is undesirable for duodenal tumours, since it has poor accuracy and may cause unexpected submucosal fibrosis. The reported overall accuracy is 68–74%, and 24.6% of suitable candidates for EMR were recommended for ESD due to endoscopic biopsy [4, 5]. Therefore, a biopsy-free preoperative diagnosis with endoscopy is necessary for SNADETs (Table 1).

**Table 1:** Accuracy, sensitivity, and specificity of preoperative endoscopic diagnosis for differentiation between C3 and C4/5 lesions

Diagnostic techniques	Endoscopic diagnostic criteria	Cases	Accuracy (%)	Sensitivity (%)	Specificity (%)	Refs.
White light endoscopy	A scoring system based on lesion diameter, color, macroscopic type, and nodularity	134	86	88	79	[6]
ME-CV	A diagnostic algorithm based on magnifying mucin phenotypes including four patterns, convoluted, leaf-like, reticular/sulciolar, and colon-like pattern	76	78.9	63.6	85.2	[7]
ME-NBI	Mucosal and vascular patterns including network, disappeared, white opaque substance and intrastructural vessels	156	72	63	76	[35]
Endocytoscopy	An endocytoscopic classification based on the structural atypia, nuclear morphology and size	93	86.7	87.7	85.4	[11]

The superficial nonampullary duodenal epithelial tumors were classified into three categories: C3 corresponds to low-grade adenoma; C4 included both high-grade adenoma and non-invasive carcinoma; C5 corresponds to invasive carcinoma

ME-CV, magnifying endoscopy with crystal violet staining; ME-NBI, magnifying endoscopy with narrow-band imaging

Tumour diameter larger than 6–10 mm, rough/nodular surface, depressed portion and erythema are considered the typical endoscopic findings of HGA and adenocarcinoma limited to the submucosal layer (SAC) [2, 5]. Recently, Kakushima et al. proposed a scoring system based on tumour diameter, macroscopic type, colour, and nodularity to differentiate between LGA and HGA or higher among SNADETs via white-light endoscopy and indigo carmine staining [6]. This scoring system predicted the histological grade with 86% accuracy if the score was greater than 3 points. The sensitivity and specificity were 88% and 79%, respectively. This scoring system is simple and may be a useful tool to improve the differential preoperative diagnosis among endoscopists with different levels of experience. Toya et al. classified magnified mucin phenotypes into four patterns, convoluted, leaf-like, reticular/sulciolar, and colon-like, and proposed a diagnostic algorithm for differentiating HGA/SAC from LGA using magnifying endoscopy with crystal violet staining [7]. This algorithm has higher accuracy than the abovementioned scoring system; its sensitivity, specificity, and accuracy were 63.6%, 85.2% and 78.9%, respectively. Yoshimura et al. utilized magnifying endoscopy combined with narrow-band imaging to explore the association between magnified mucosal and vascular patterns and histological grades of SNADETs. They found that a network vascular pattern and an obscure mucosal pattern were more often detected in the final histology of HGA/SAC [8]. Recently, mucin phenotypes of SNADETs have been classified into three groups, intestinal, gastric and gastrointestinal, based on immunohistopathological studies. The gastric phenotype has a significantly higher histological grade (HGA/SAC) and is associated with worse prognosis than the intestinal phenotype [1].

Microscopic endoscopy, including endocytoscopy and confocal laser endomicroscopy, has been developed to diagnose SNADETs [9, 10]. Microscopic endoscopy can observe lesions at the cellular level during endocytoscopy in real time without histopathological biopsy. Muramoto et al. conducted a prospective study and established a new classification system for the diagnosis of SNADETs based on endocytoscopic findings, such as the degree of structural atypia and the nuclear morphology and size of the lesions, with 87.7% sensitivity and 85.4% specificity for the preoperative diagnosis of HGA/SAC [11]. Microscopic endoscopy is an ideal technique to predict the histopathology of SNADETs in real time and guide subsequent appropriate therapeutic strategies.

## Endoscopic treatment modality for SNADETs

Presently, several treatment options are available for SNADETs, ranging from the least invasive cold snare polypectomy (CSP) to surgical resection [12, 13]. Previous studies have reported that duodenal intramucosal carcinoma showed no lymph node metastasis. Therefore, adenoma or clinical intramucosal carcinomas are suitable candidates for local resection (Table 2).

**Table 2:** Indication, advantages and disadvantages of each endoscopic treatment for superficial nonampullary duodenal epithelial tumors

Method	Advantages	Disadvantages	Indications
C-EMR	Moderate feasibility Moderate safety	Low en bloc resection rate with lesion > 20 mm	C3: > 10 mm C4/5: < 20 mm without submucosal invasive
Cold snare polypectomy	Fast Simple Less thermal damage	Incomplete excision, Inaccurate excision margin	C3: < 10 mm
Underwater EMR	Lower risk of perforation and safer than EMR	Low en bloc rate for lesions > 20 mm	C3: > 10 mm C4/5: < 20 mm without submucosal invasive
ESD	High en bloc rate	High complication rate Technically challenging	C4/5: < 30 mm without submucosal invasive
Modified ESD			
The pocket-creation method	Less complication rate Better scope control during ESD	High cost	C4/5: < 30 mm without submucosal invasive
Water pressure method	Shortens procedure times	Special device	C4/5: < 30 mm without submucosal invasive
Laparoscopic endoscopic collaborative surgery	High en bloc rate Less complication rate	Technically challenging High cost Lack of long-term data	C4/5: 20–40 mm and more than 10 mm from the papilla, without submucosal invasive

The superficial nonampullary duodenal epithelial tumors were classified into three categories: C3 corresponds to low-grade adenoma; C4 included both high-grade adenoma and non-invasive carcinoma; C5 corresponds to invasive carcinoma

C-EMR, conventional endoscopic mucosal resection; EMR, endoscopic mucosal resection; ESD, endoscopic submucosal dissection

EMR using a snare for SNADETs was reported in 1997, and the duodenal EMR en bloc resection rate was 80–90% for lesions smaller than 20 mm and 30–40% for lesions over 20 mm [14]. Local recurrence was reported in 5–37% of cases after piecemeal EMR that were retreated by EMR or argon plasma coagulation, because most were adenomatous tissue [15, 16]. Regarding its safety, the intra-EMR perforation rate was 0–2%, delayed perforation occurred in 0–4% of cases, and the incidence of bleeding was reported in approximately 5–15% of cases [17]. Recently, safer resection methods, such as CSP and underwater EMR, have been applied for SNADETs [18, 19]. CSP is an easy and quick method for small colorectal polyps and performed well in small duodenal lesions less than 10 mm without electrocautery or submucosal injection. Furthermore, CSP has the same en bloc resection rate as hot snare polypectomy and has a lower risk of perforation and bleeding than hot snare polypectomy or EMR. However, studies on colonic polyps showed that only 2% of CSP samples included the submucosa [20]. Therefore, currently, CSP is unsuitable for lesions that require resection of submucosal tissue. Underwater EMR was reported for use with duodenal adenoma in 2013, a procedure that was originally developed for colorectal polyps in 2012. Underwater EMR is safe and enables easy resection of small SNADETs and is expected to reduce the risk of adverse events. In this method, lesions, including flat or sessile lesions, float up in a way similar to protruded lesions, and duodenal angles become obtuse, which facilitates easy snaring of lesions underwater. In addition, underwater EMR decreases thermal damage to the muscle layer, and mucosal defects are easy to close with endoclips, because the surrounding duodenal mucosa is soft, because no injections are used. It has been reported that the complete resection rate of this method for SNADETs smaller than 20 mm was 79%, and no perforation was reported in any of the cases [20, 21]. Briefly, although the en bloc resection rate of EMR, including CSP and underwater EMR, is insufficient for lesions over 20 mm, the safety profiles of these procedures are acceptable, and currently, EMR using a snare is the standard endoscopic therapy for small SNADETs [22].

ESD was approved for use in the duodenum in 2006. The procedure results in a higher en bloc resection rate and lower local recurrence rate than those seen with EMR and is used for duodenal lesions that require complete resection or lesions that cannot be resected by EMR, such as those that are poorly lifted after injection [23]. However, duodenal ESD is technically demanding and has a high rate of complications due to thin duodenal muscularis propria, poor intraoperative endoscopic view, and post-ESD ulcer exposure to pancreatic and bile juices. The rates of intraoperative perforation and delayed perforation after ESD were reported to be 9–39% and up to 9%, respectively [12]. Several new and safe ESD strategies have been developed and are expected to overcome the difficulty of current ESD procedures and reduce the risk of adverse events. “The pocket-creation method”, which involves making a submucosal pocket with a minimal initial mucosal incision after submucosal dissection of the same area, provides adequate space for the fixation of the endoscope and good observation of the targeted submucosa during ESD. Encouraging results of duodenal ESD using this method have been reported with a 100% en bloc resection rate and 4% perforation rate [24]. The “water pressure method” is another unique technique that reduces the intraprocedural perforation risk and shortens procedure times. In this method, the waterjet function of the endoscope is used, and the water pressure improves the view of the submucosal layer underwater during ESD [25]. Another approach utilizes double-balloon endoscopes, which were originally developed for use in the small intestine, during duodenal ESD to reduce the redundant angles between the stomach and duodenum [26]. In addition, this procedure aids in endoscopic control and improves the endoscopic view of the muscularis propria layer.

Closure of post-ESD ulcers is another strategy to enhance the safety of duodenal ESD, including the polyglycolic acid (PGA) shielding method [27, 28], closure with clips or string alone or in combination [29], and closure using over-the-scope clips (OTSCs) [30]. PGA sheets and fibrin glue can remain in place for more than a week. Closure with a combination of clips and string may be simpler to perform and provide a stronger closure than that obtained using clips alone and could prevent early displacement of the clips. In addition, OTSCs, besides being haemostatic devices, are also used to close duodenal mucosal wounds because of their strong holding and grasping forces. Furthermore, OTSCs can remain on the ulcer bed for several months. Reported rates of delayed perforation and bleeding in the complete closure and incomplete/no closure groups were 1.7% and 10.5% and 0% and 10.5%, respectively [31].

Laparoscopic and endoscopic cooperative surgery (LECS) was originally developed for gastric submucosal tumours as a less invasive surgical procedure, but it has also been applied to duodenal tumours at an early stage to ensure sufficient resection of the lesions and decrease the risk of complications [32]. In LECS for SNADETs, the post-ESD mucosal defect is tightly reinforced after laparoscopic suturing of the duodenal wall using seromuscular sutures from the extraluminal side. Encouraging results have been reported for SNADETs approximately 30 mm in size and located more than 10 mm away from the ampulla of Vater [32]. However, we should be aware that lesions on the inner side of the duodenum behind the pancreatic head parenchyma are not candidates for this method due to the risk of postoperative stricture and pancreatic fluid fistula [33, 34]. Moreover, it is necessary to preoperatively exclude its use in submucosal invasive adenocarcinomas with lymph node metastasis, because additional surgery would be difficult due to alteration and adhesion of anatomy and lymphatic flow casing caused by incomplete LECS. Finally, the reported delayed perforation rate is not 0%, even if the mucosal defect after ESD is closed by laparoscopic suturing [35].

## Conclusions

Duodenal tumours are rare but severe cancers among small intestine cancers. Local resection treatment should be selected based on the malignant potential, location, and size of the tumour as well as efficacy and safety. At present, ESD for SNADETs is technically challenging and has a notably high risk of adverse events, which limits the clinical applicability of endoscopic en bloc resection in the duodenum. However, the safety and R0 resection rate have improved with progress in new techniques and devices. Prospective studies are necessary to evaluate the disease-free survival of patients with SNADETs who underwent piecemeal or en bloc resection and to determine whether ESD could become a part of the standard of care for these patients.

## Data Availability

Data sharing is not applicable to this article as no data sets were generated or analyzed during the current study.

## Abbreviations

SNADETs: Superficial nonampullary duodenal epithelial tumours
LGA: Low-grade adenoma
HGA: High-grade adenoma
EMR: Endoscopic mucosal resection
ESD: Endoscopic submucosal dissection
SAC: Adenocarcinoma limited to the submucosal layer
CSP: Cold snare polypectomy
PGA: Polyglycolic acid
OTSCs: Over-the-scope-clips
LECS: Laparoscopic and endoscopic cooperative surgery
